# Acute Paracoccidioidomycosis Due to *Paracoccidioides brasiliensis* S1 Mimicking Hypereosinophilic Syndrome with Massive Splenomegaly: Diagnostic Challenge

**DOI:** 10.1371/journal.pntd.0004487

**Published:** 2016-04-07

**Authors:** Priscila Marques de Macedo, Luã Cardoso de Oliveira, Dayvison Francis Saraiva Freitas, Jaqueline Abel da Rocha, Andrea D’Ávila Freitas, Marcio Nucci, Rosely Maria Zancopé-Oliveira, Rodrigo Almeida-Paes, Antonio Carlos Francesconi do Valle

**Affiliations:** 1 Infectious Dermatology Clinical Research Laboratory, Evandro Chagas National Institute of Infectious Diseases, Fiocruz, Rio de Janeiro, Brazil; 2 Mycology Laboratory, Evandro Chagas National Institute of Infectious Diseases, Fiocruz, Rio de Janeiro, Brazil; 3 Nosocomial Infection Surveillance and Control Program, Central Hospital of Military Police (HCPM), Rio de Janeiro, Brazil; 4 Department of Inpatient Health Care, Evandro Chagas National Institute of Infectious Diseases, Fiocruz, Rio de Janeiro, Brazil; 5 Department of Internal Medicine, Universidade Federal do Rio de Janeiro (HUCFF, UFRJ), Rio de Janeiro, Brazil; Hospital Infantil de Mexico Federico Gomez, UNITED STATES

## Case Presentation

An 18-year-old male student presented to the hospital complaining of abdominal pain, fever, and 4 kg weight loss in the preceding 2 months. Six weeks before the beginning of the symptoms, the patient reported a cliff fall when he was practicing longboard at Alto da Boa Vista, an urban forest area in Rio de Janeiro, Brazil. For almost 30 minutes he remained trapped in the woods, inhaling dust of the local soil. There was no report of preceding travels or rural activities. Physical examination showed mild hepatosplenomegaly without peripheral lymphadenopathy, and vital signs were normal. Laboratory analyses revealed leukocytosis 35,670/mm^3^ (Reference value [RV]: 4,200–9,000/mm^3^) with predominance of eosinophils 72% (RV: 1%–7%); hemoglobin 12.6 g/dL (RV: 13–18 g/dL); platelet count 198,000/mm^3^ (RV: 150,000–450,000/mm^3^); normal serum biochemistry, parasitological analysis of the feces, and chest X-ray; and negative anti-HIV ELISA serology. Idiopathic hypereosinophilic syndrome was suspected, and he was started on empirical methylprednisolone (1 mg/kg/d) and sulfamethoxazole/trimethoprim (SMZ/TMP 800/160 mg b.i.d. three times a week). After 7 days the patient improved, with defervescence and a progressive reduction in white cells and eosinophil counts observed within a month. Nevertheless, the attempt of gradual withdrawal of corticosteroids to 5 mg (and also suspension of SMZ/TMP) resulted in deterioration in the general clinical condition, recurrence of fever, and an enlargement of the spleen noticed 14 days after this dosage was reached. Bone marrow biopsy was performed and showed, in hematoxylin–eosin staining, rounded fungal structures with multiple peripheral budding, suggestive of *Paracoccidioides* spp. The Ouchterlony double radial immunodiffusion test (ID) for paracoccidioidomycosis (PCM) was positive. Computerized tomography of the abdomen showed an enlarged spleen ranging nearly 30 cm with foci of ischemia. Liposomal amphotericin B was given (3 mg/kg/d) for 32 days, after which SMZ/TMP was introduced (800/160 mg t.i.d).

**Fig 1:**
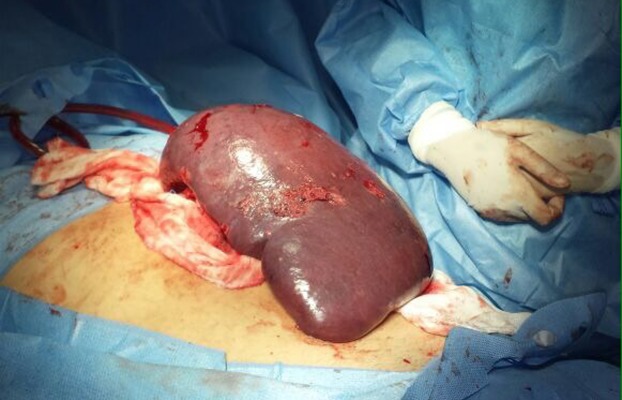
Massive splenomegaly ranging 30 cm with necrotic foci after splenectomy performed in a patient with disseminated and acute paracoccidioidomycosis.

Because of massive splenomegaly, progressive decrease in platelet counts (up to 30,000/mm^3^), and consideration of the spleen as a focus of infection, a splenectomy was performed (Fig 1). Samples of the spleen were sent for microbiological analyses, and filamentous colonies with a grey compact folded aerial mycelium started to grow after 3 weeks on Potato Dextrose Agar, successfully converting into the yeast phase after incubation at 37°C on Fava-Netto’s Agar medium. After splenectomy and 5 months taking SMZ/TMP, the patient recovered from fever and other serious health conditions. Leucocytes, eosinophil, and platelet counts reached a normal range (8,850/mm^3^, 1%, and 422,000/mm^3^, respectively). Patient is still undergoing treatment.

## Ethical Aspects

This work was approved by the Research Ethics Committee (CEP) Fiocruz, CAAE 42590515.0.0000.5262. Patient has signed the consent form for publication.

## Molecular Characterization of the Isolate

Genomic DNA was extracted from the yeast phase according to Ferrer et al. [1]. The identity of the isolate was evaluated using 100 ng of genomic fungal DNA for the amplification of the nucleotide sequence of two partial protein-encoding genes: *arf* (ADP rybosilation factor) and *gp43* loci [2]. The following primers (1 μL) were used in a 10 μM concentration: ARF-F 5’TCTCATGGTTGGCCTCGATGCTGCC3’ and ARF-R 5’GAGCCTCGACGACACGGTCACGATC3’; and gp43-E2F 5’CCAGGAGGCGTGCAGGTGTCCC3’ and gp43-E2R 5’GCCCCCTCCGTCTTCCATGTCC3’, as previously described by Matute et al. [3]. Polymerase chain reaction (PCR) cycles were performed as follows: pre-denaturation (94°C for 2 minutes); amplification in 35 cycles of 94°C, denaturation for 30 seconds, annealing 60°C (both *arf* and *gp43* loci) for 30 seconds, and 68°C extension for 1 minute; with a final extension of 5 minutes at 68°C. Forward and reverse DNA segments were sequenced (Sanger Method) using the above listed primers. Sequences from both DNA strands were generated, edited with the Sequencher version 4.6 software package (Gene Codes Corporation, United States), and aligned by means of the Mega version 6.0 software. The sequences of our isolate (available in GenBank: KU042924, KU042925 for *gp43* and *arf* loci, respectively) were compared by BLAST (Basic Local Alignment Search Tool) with sequences from isolates belonging to the *Paracoccidioides brasiliensis* complex previously deposited at GenBank (http://www.ncbi.nlm.nih.gov/genbank/) by Matute et al. Our sequences presented 100% similarity with *P*. *brasiliensis* S1.

## Case Discussion

PCM is considered a neglected infectious disease, despite being the first cause of death among all systemic mycoses in immunocompetent patients and eighth among chronic or recurrent infectious and parasitic diseases in Brazil [4,5]. In this report, we describe the diagnostic challenge of a severe and atypical presentation caused by *P*. *brasiliensis* S1 cryptic species identified through sequencing of the genes *arf* and *gp43*.

Cryptic species in the genus *Paracoccidioides* were first described by Matute et al. through multilocus sequence typing (MLST) and comprise a group with more genetic similarity patterns: *Paracoccidioides brasiliensis* S1, PS2, PS3, PS4, and a species with a well distinguished genetic profile: Pb01-like, thereafter named *Paracoccidioides lutzii* in tribute to Adolpho Lutz, who first described the disease [2,3,6–9].

Recent studies have shown these species have different patterns of geographic distribution and may be related to different clinical presentations, sensitivity in the ID serological tests, and prognosis [2,10–12]. Until now, *P*. *lutzii* has been supposed to be associated with more serious clinical conditions such as lymphoabdominal and fatal fungemia [13]. This species seems to be limited to the central region of Brazil, although few cases have been reported in other regions [11,14].

This is the first case described with molecular identification of PCM in the state of Rio de Janeiro, an important endemic area for PCM in Brazil. A remarkable finding of our case is that the patient never traveled to rural areas, since PCM is not common in urban centers. Finally, another interesting finding was the significant eosinophilia.

Hypereosinophilic syndrome is a heterogeneous group of myeloproliferative disorders characterized by the presence of marked peripheral blood eosinophilia (absolute count > 1,500/mm^3^), tissue eosinophilia, or both, resulting in a wide variety of clinical manifestations [15]. Secondary causes of eosinophilia must be ruled out. Eosinophil counts in acute PCM are usually high because of IgE production and other cytokines of Th2 response to the parasite [16]. Disseminated and acute types of PCM with more than 30% eosinophils and massive splenomegaly without lymphadenopathy are very uncommon.

It is relevant to mention that PCM is acquired through inhalation of infectious propagules, which then lodge in the alveoli, from which they can spread to many organs, particularly the mononuclear phagocyte system [17]. In the case herein described, bone marrow and spleen were critically affected. Corticotherapy may have contributed to severe presentation in this case.

So far, *P*. *brasiliensis* S1 species had never been associated with severe clinical forms of PCM with a favorable outcome. In fact, only a few case reports of PCM included molecular identification. Our report illustrates the difficulty in recognizing PCM presenting with hypereosinophilia and massive splenomegaly and highlights the need for more studies evaluating different types of clinical presentations, since pathogenesis of PCM seems to depend not only on virulence of cryptic species of fungal complex but also on the immune status of the host.

**Key Learning Points**

- Paracoccidioidomycosis must be considered as a differential diagnosis in cases of hypereosinophilia in endemic areas of this mycosis.
- Massive splenomegaly was a great point of challenge for diagnosis.
- The lack of lymphadenopathy in the physical examination and an urban forest as probable source of infection are also remarkable challenging points for diagnosis.
